# The Role of Ethylene in Plant Adaptations for Phosphate Acquisition in Soils – A Review

**DOI:** 10.3389/fpls.2015.01224

**Published:** 2016-01-20

**Authors:** Günter Neumann

**Affiliations:** Nutritional Crop Physiology, Institute of Crop Science, University of HohenheimStuttgart, Germany

**Keywords:** ethylene, root growth, root morphology, phosphate deficiency, phosphate acquistion

## Abstract

Although a role of ethylene in the regulation of senescence and plant stress responses in general has a long history, a possible involvement in the regulation of adaptive responses to nutrient deficiencies has been mainly investigated since the last two decades. In the case of plant responses to phosphate (P_i_) starvation, ethylene was identified as a modulator of adaptive responses in root growth and morphology. The molecular base of these adaptations has been elucidated in supplementation studies with ethylene precursors and antagonists, as well as analysis of mutants and transgenic plants with modified ethylene biosynthesis and responsiveness, using mainly *Arabidopsis thaliana* as a model plant. However, increasing evidence suggests that apart from root growth responses, ethylene may be involved in various additional plant adaptations to P_i_ limitation including P_i_ mobilization in the rhizosphere, P_i_ uptake and internal P_i_ recycling. The ethylene-mediated responses are frequently characterized by high genotypic variability and may partially share common pathways in different nutrient limitations.

## Introduction

**FIGURE 1:**
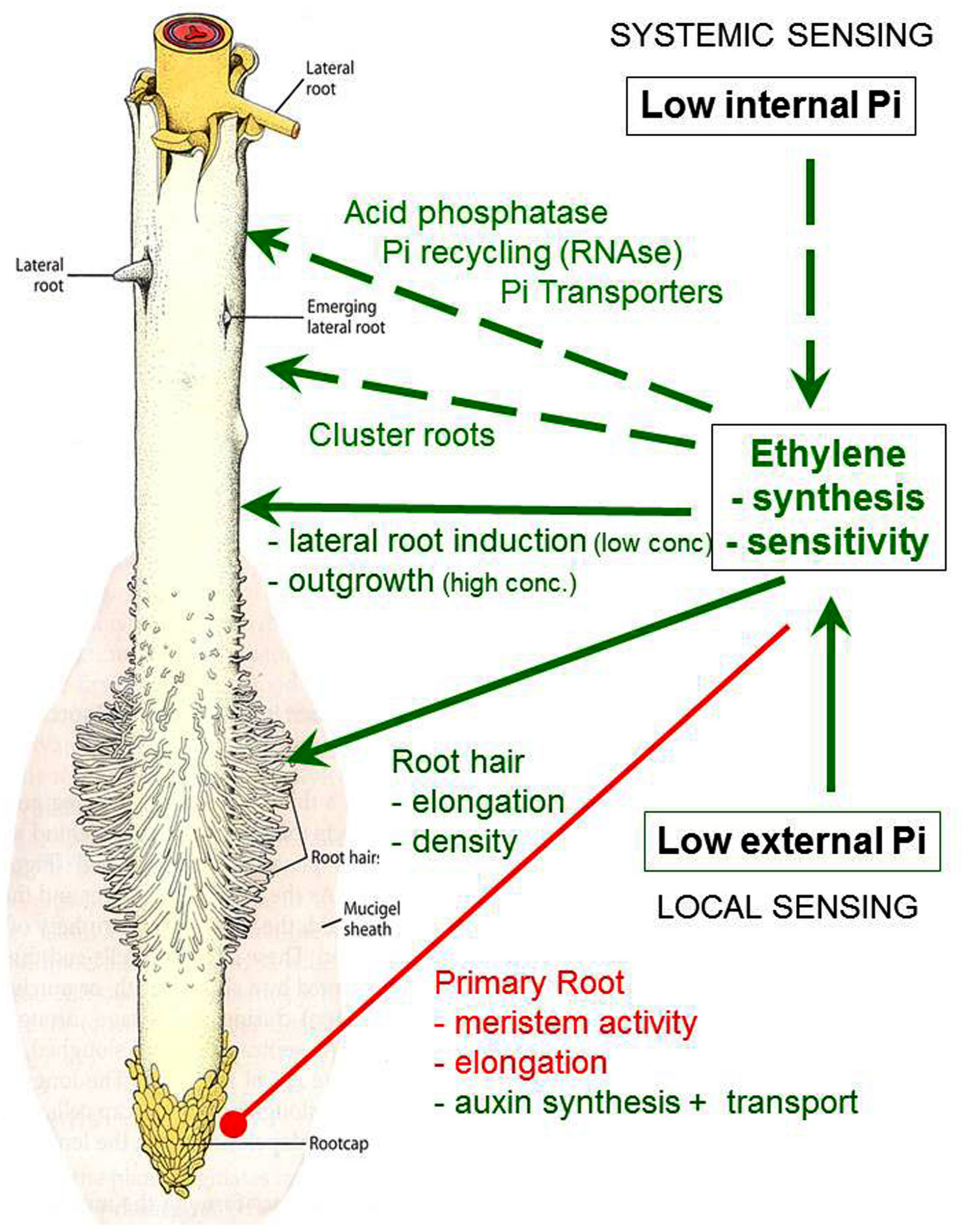
**Adaptive responses to P_i_ limitation in plant roots modulated by ethylene.** Green: stimulation; red: inhibition.

**FIGURE 2:**
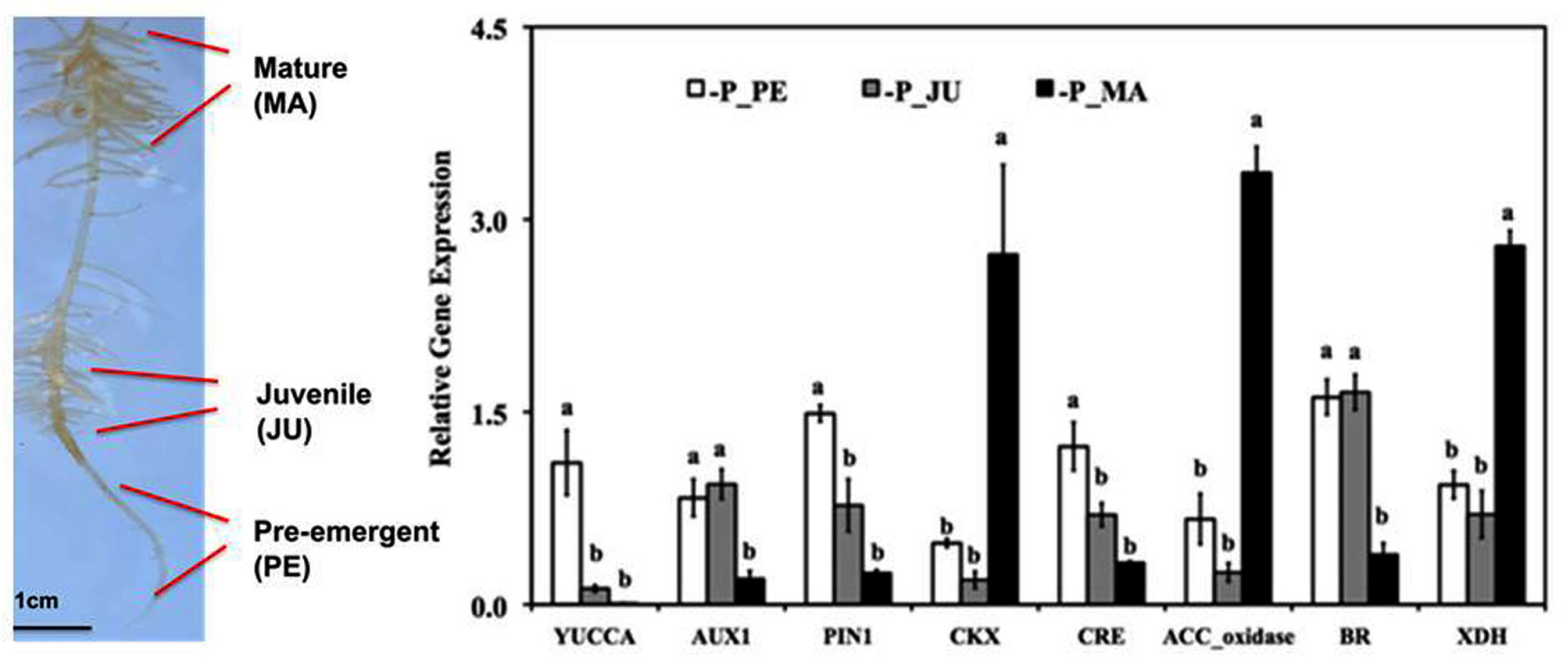
**Expression pattern of hormone-related genes during cluster root (CR) development in Lupinus albus grown for 20 days without P_i_ supply and evaluated by quantitative real time PCR (RT-qPCR).** Genes are involved in auxin biosynthesis (YUCCA), auxin transport (AUX1, PIN1), cytokinin degradation (CKX), cytokinin receptors (CRE), ethylene biosynthesis (ACC_oxidase), brassinosteroid biosynthesis (BR), and RNA degradation associated with nitric oxide formation (XDH). The gene expression level is indicated relative to reference genes. Data represent means ± SE (*n* = 2–3). Different lowercase letters denote significant differences (*P* < 0.05). PE, pre-emergent stage; JU, juvenile root clusters; MA, mature root clusters (modified after Wang et al., 2014b). The photo shows the different developmental stages of CRs used for RNA isolation: PE, pre-emergent stage; JU, juvenile cluster; MA, mature cluster.

Among the wide range of phosphorus (P) forms in soils, inorganic phosphate anions (P_i_) are taken up exclusively by plant roots. However, due to limited solubility, P is the macronutrient with the lowest plant availability in soils. Even in well-fertilized soils, on average only 20% of the fertilizer input are utilized by plants since the majority of fertilizer P_i_ is prone to P_i_ fixation and incorporation into organic P_i_ forms comprising 20–80% of total soil P. (Richardson, 1994; Holford, 1997). Therefore, higher plants are strongly dependent on specific adaptations to acquire P_i_ in sufficient amounts. Adaptive responses toward improved spatial P_i_ acquisition comprise stimulation of root growth, increased formation of fine root structures (lateral roots, root hairs) (**Figure 1**), preferential root development in the top soil with the highest P content (Lynch and Brown, 2001) or stimulation of lateral root growth in nutrient patches rich in P and also N (Forde and Lorenzo, 2001). Modifications of the rhizosphere chemistry, such as rhizosphere acidification, secretion of organic metal-chelators (carboxylates, phenolics) and phosphohydrolases (acid phosphatase, phytase) increase the solubility and plant availability of P_i_ in the rhizosphere (Neumann and Römheld, 2002, 2007). The formation of so-called cluster roots (CR; **Figure 2**) within the Proteaceae, Casuarinaceae, Myrtaceae, and Fabaceae (Dinkelaker et al., 1995; Neumann and Martinoia, 2002), or dauciform roots in Cyperaceae (Playsted et al., 2006) are among the most specialized root-morphological adaptations to promote the secretion of Pi-mobilizing root exudates. The expression of high affinity P_i_ uptake systems provides the ability for efficient exploitation of the rhizosphere solution even at low P_i_ levels, hardly exceeding concentrations of 10 μM (Bieleski, 1973) even in well-fertilized soils. Also symbiotic associations with mycorrhizal fungi are frequently established as adaptive responses for improved spatial (arbuscular and ectomycorrhizal fungi) and chemical acquisition (mainly ectomycorrhizal fungi) of soil P forms (Neumann and Römheld, 2002).

Among the wide range of regulatory factors involved in the induction of adaptive responses to P_i_ limitation, there is increasing evidence that these processes are modulated also by ethylene as important regulator. In many studies, the role of ethylene has been investigated by exogenous application of precursors and antagonists of ethylene synthesis and signal transduction and by expression analysis of genes involved in ethylene biosynthesis, signaling and ethylene responses. Other strategies comprise the analysis of mutants and transgenic plants with modified synthesis, signaling and reception of ethylene, most frequently using *Arabidopsis thaliana* as model plant (Nagarajan and Smith, 2011).

## Ethylene and Root Growth Responses

The involvement of ethylene in regulation of root growth has been postulated already in early studies by Chadwick and Burg (1967) on root geotropism and Smith and Russell (1969) on root growth responses under oxygen limitation, including also interactions with auxin (Chadwick and Burg, 1967, 1970). Meanwhile it is generally accepted that ethylene influences root growth in a biphasic manner with stimulatory effects, e.g., on lateral root formation induced by low ethylene concentrations, triggering both, synthesis and signaling of auxins, as indicated by analysis of *Arabidopsis* mutants affected in auxin signaling and ethylene-induced auxin synthesis (Stepanova et al., 2005; Ivanchenko et al., 2008). The ethylene-induced modifications of auxin synthesis and transport contribute to the formation of auxin gradients necessary for the induction of lateral root primordia in the pericycle opposite the prototoxylem poles (Fukaki and Tasaka, 2009).

By contrast, high ethylene concentrations exert inhibitory effects on lateral root formation, as demonstrated by Negi et al. (2008), showing that both, overproduction of ethylene by high external application of the ethylene precursor 1-aminocyclopropane-1-carboxylic acid (ACC) or by the *eto1* mutation, inhibited lateral root formation in *Arabidopsis*. On the other hand, lateral root formation was stimulated in the *etr1* (ethylene triple response1) or *ein2* (ethylene insensitive2) mutations, blocking ethylene responses (Negi et al., 2008). Similar to lateral root formation promoted by low levels of ethylene, ethylene/auxin interactions seem to be involved also in the inhibitory effects on root growth induced by high ethylene concentrations, stimulating both, acropetal and basipetal auxin transport with involvement of the AUX1 influx carrier as indicated by an ethylene-insensitive *aux1-7* mutant of *Arabidopsis* (Negi et al., 2008), as well as PIN3 and PIN7 eﬄux transporters (Lewis et al., 2011). The ethylene-mediated stimulation of auxin transport may inhibit lateral root formation by a reduction of auxin accumulation in the protoxylem pericycle, required for initiation of lateral root primordia (Fukaki and Tasaka, 2009). Interestingly, high ethylene concentrations exerted inhibitory effects on formation of new lateral root primordia but stimulated outgrowth of already existing primordia (Ivanchenko et al., 2008). In primary roots of *Arabidopsis*, also a massive ethylene-induced stimulation of auxin synthesis in the root tip has been observed (Ruzicka et al., 2007; Swarup et al., 2007) with inhibitory effects on root growth, which may at least partially be attributed to a reduced extensibility of the cell wall due to inhibition of the auxin-dependent plasmalemma H^+^-ATPase and formation of reactive oxygen species (ROS), promoting cross-linking of cell walls by hydroxyproline-rich glycoproteins in response to high auxin concentrations. Increased ethylene levels are also able to affect the activity of the primary root meristem, probably by interaction with jasmonic acid (Chacon-Lopez et al., 2011), inducing a determinate developmental program with arrested cell division and promotion of cell differentiation.

## Adaptive Responses to P_i_ Limitation – Spatial P_i_ Acquisition

**FIGURE 3:**
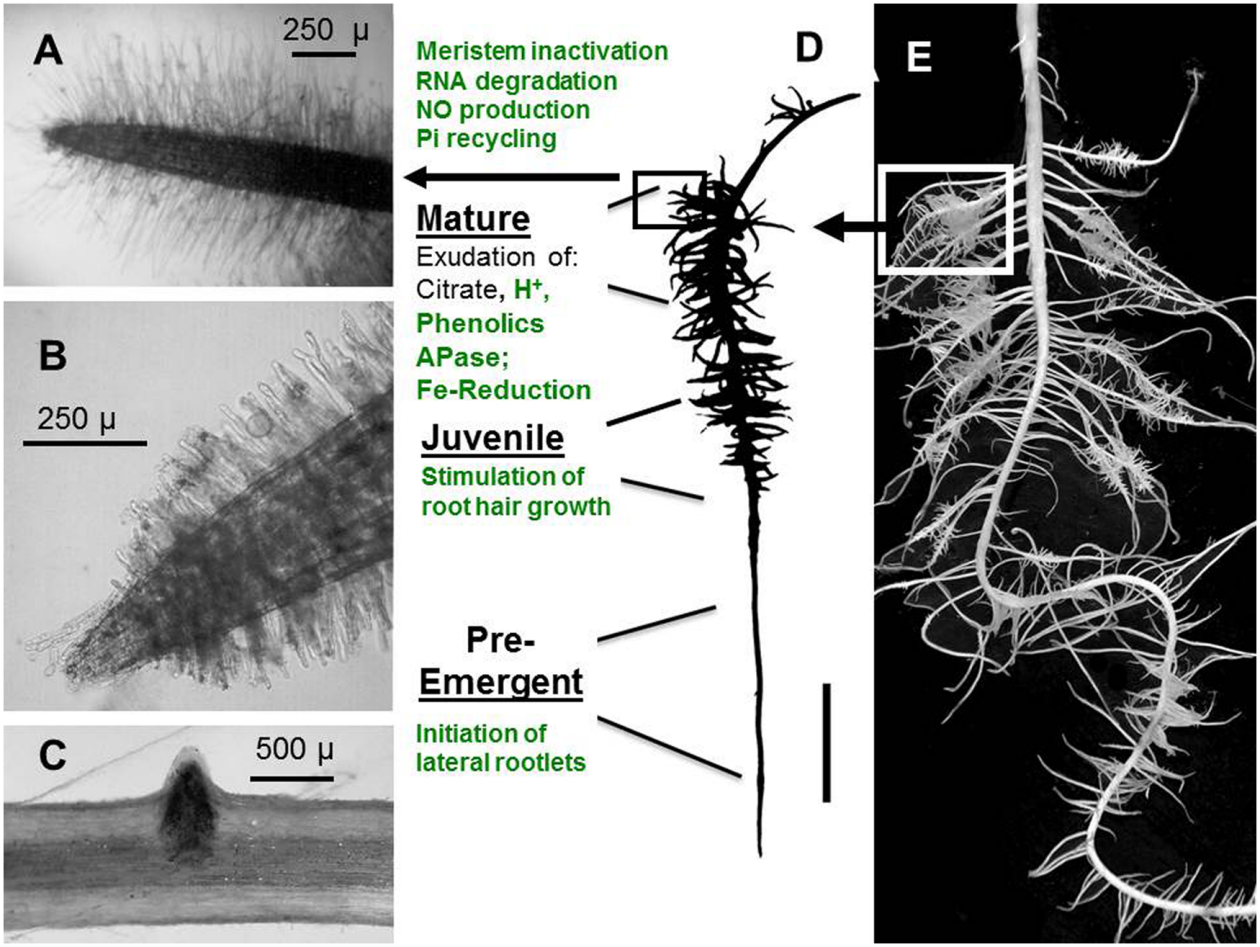
**Stages of CR development in P_i_-deficient white lupin (*Lupinus albus* L.).** Characteristic processes in the different root zones potentially modulated by ethylene are marked in green. **(A)** Single second order lateral rootlet of a MA root cluster, densely covered with root hairs. **(B)** Single lateral rootlet of an outgrowing juvenileJU root cluster with growing root hairs. **(C)** Outgrowth of a lateral rootlet primordium. **(D)** Root clusters in different developmental stages along a first-order lateral root; **(E)** Root system of P_i_-deficient white lupin with CRs development. PE, pre-emergent stage; JU, juvenile cluster; MA, mature cluster. (Figure modified after Wang et al., 2014b).

Measurements of ethylene production, inhibitor studies (Borch et al., 1999; Lynch and Brown, 2001; Li et al., 2009), analyses of mutants in gene expression of the ethylene bio-synthetic pathway (Tsuchisaka and Theologis, 2004; O’Rourke et al., 2013; Wang et al., 2014a) revealed promotion of ethylene synthesis and/or enhanced ethylene sensitivity (**Figure 1**), induced by P_i_ limitation in higher plants (He et al., 1992; Kim et al., 2008). These responses seem to be expressed in a highly tissue-specific and developmental stage-dependent manner. Accordingly, Kim et al. (2008) reported up-regulation of ethylene production in shoots but not in roots of P_i_-deficient tomato and no effects in Petunia with the conclusion that modifications in ethylene sensitivity are more important in latter cases. As another example, Wang et al. (2014a) recorded up-regulation of the ethylene biosynthesis gene encoding ACC oxidase in 1–2 cm sub-apical lateral root zones just prior emergence of secondary laterals during CR development in Pi-deficient white lupin (*Lupinus albus* L.). Gene expression of ACC oxidase declined after outgrowth of the secondary laterals, followed by a massive increase again during further development of the root clusters (**Figure 3**).

The P_i_ deficiency-induced changes in ethylene accumulation and ethylene responsiveness are involved in adaptive modifications of root growth toward improved P acquisition. Typical patterns comprise a reduction of primary root growth, associated with an increase in lateral root development (**Figure 1**) promoting the development of a shallower root system for exploitation of top soil layers with the highest P availability (Linkohr et al., 2002; Lopez-Bucio et al., 2002; Sanchez-Calderon et al., 2006; Svistoonoff et al., 2007). Similarly, the angle of basal lateral roots in common bean can be modulated by increased sensitivity to ethylene in response to low P_i_ supply, to direct lateral root development into the upper soil layers (Basu et al., 2007). However, the described ethylene-mediated root growth responses to P limitation cannot be generalized and exhibit high genotypic variability. Testing 73 ecotypes of *Arabidopsis thaliana* revealed P deficiency-induced inhibition of primary root elongation only for 50% of the accessions (Chevalier et al., 2003). A survey of 14 dicots and monocots in hydroponics showed that all tested species had the same degree of primary root elongation independent of the P_i_-nutritional status (Narayanan and Reddy, 1982), and many plant species even exhibit root elongation under low-P_i_ conditions (Niu et al., 2012). Also the formation of shallower root systems in common bean is a heritable trait with genotypic variability, which has already been exploited for breeding programs to promote top soil foraging for improved P_i_ acquisition (Lynch and Brown, 2012).

One of the earliest detectable modifications of root morphology in response to P_i_ starvation is an increased number and length of root hairs (Bates and Lynch, 2001; Ma et al., 2001; Jain et al., 2007) as an important adaptation for improved spatial acquisition of available P_i_ in the rhizosphere (**Figures 1** and **2**) with particular importance for plant species unable to form mycorrhizal associations. Accordingly, length of root hairs was inversely correlated with the degree mycotrophy in different plant species (Schweiger et al., 1995). However, due to secretory properties (protons, organic metal chelators, mucilage) and surface extension, the length and the density of root hairs also determines root soil contact and chemical modifications of the rhizosphere toward improved solubilization of nutrients (Playsted et al., 2006; Haling et al., 2013; Abrahão et al., 2014).

Based on the observation that in contrast to Fe deficiency, the number of root hairs in *Arabidopsis* under P_i_ limitation was not affected by application of ethylene anatagonists and also not in the ethylene-insensitive *ein2* and the ethylene-resitant *etr1* mutants, Schmidt and Schikora (2001) concluded that the development of extra root hairs in response to P_i_ limitation does not appear to require ethylene signaling. However, treatments with the ethylene precursor ACC promoted root hair elongation, which was inhibited by ethylene antagonists (Zhang et al., 2003). Moreover, root hair length was reduced in various ethylene-response mutants as compared with the wild type under P limitation but not with sufficient P_i_ supply (Cho and Cosgrove, 2002; Zhang et al., 2003), suggesting that ethylene is involved in the regulation of P_i_ deficiency-induced root hair elongation (**Figure 1**). Moreover, ethylene increased also the density of root hairs by shortening trichoblast cells to increase the number of H cells per unit root length (Zhang et al., 2003). Apart from the hormone-dependent metabolic regulation, root hair development in response to P_i_ limitation also shows marked genotypic variations and improved P acquisition in cultivars with long root hairs has been documented for barley (Gahoonia et al., 1997; Haling et al., 2013) and *Phaseolus vulgaris* where a combination of ethylene-modulated root traits, such as long root hairs and a shallow root system was particularly efficient (Miguel et al., 2015).

Interestingly, the ethylene mediated responses of root growth to P_i_ limitation as described so far, seem to be largely independent from a low P-nutritional status of the plant as a systemic signal, and a low P_i_ level in the external rooting medium seems to be sufficient for the induction (Thibaud et al., 2010; Nagarajan and Smith, 2011). The local sensor is currently unknown but it seems to be plausible that high affinity P transporters, located in the plasma membrane of epidermal cells in roots and root hairs, could express a double function as transporters and receptors (transceptors) as already shown for the yeast Pho84p high-affinity P_i_ transporter (Popova et al., 2010) or the *Arabidopsis* nitrate transporter CHL1/NRT1.1 with functions as transporter and sensor for nitrate in the external medium (Ho et al., 2009). The low P_i_ status of the rooting medium is most probably sensed in the apoplast of the primary root tip (Svistoonoff et al., 2007) and a P5-type ATPase (PDR2) interacting with the SCARECROW transcription factor and multi-copper oxidases (LPR1/LPR2) in the ER of the root tip meristem have been characterized as components of the sensing system. After sensing the local P_i_ status at the primary root tip, the information of P_i_ depletion at the roots is translocated via xylem transport to the shoot and may involve P_i_, strigolactones, and cytokinins as signal molecules (recently reviewed by Chiou and Lin (2011) and Zhang et al. (2014).

## Adaptive Responses to P_i_ Limitation – P_i_ Mobilization

Apart from functions in adaptive modulation of root morphology and root architecture for improved spatial acquisition of available soil P_i_, there is also increasing evidence for a role of ethylene in root-induced adaptations to increase the chemical availability of P_i_ in the rhizosphere. A large proportion of soil P_i_ (up to 80 %) is usually sequestered in organic binding forms, requiring mineralization by enzymatic hydrolysis prior to plant uptake (Richardson, 1994; Holford, 1997). Accordingly, both, soil microorganisms and plant roots are able release phosphohydrolases (e.g., acid phosphatases, alkaline phosphatases, phytases, nucleases) to acquire or recycle P_i_ from organic binding forms. Particularly root secretion of acid phosphatases is stimulated as a response to P_i_ limitation in many plant species (Neumann and Römheld, 2007) and ethylene signalling seems to be involved in the up-regulation of intracellular and secretory acid phosphatases (**Figure 1**), both, at the level of transcription and enzyme activity as indicated by precursor/inhibitor experiments and analysis of the ethylene insensitive *ein2-5* and the ethylene-overproducing *hps2* mutant (Lei et al., 2011; Li et al., 2011).

In contrast to the adaptive responses in root growth, the up-regulation of acid phosphatases is induced systemically by a low internal P_i_ nutritional status of the plant. Other systemic, potentially ethylene-mediated responses comprise the up-regulation of ribonuclease genes (RNS1), intracellular acid phosphatases (ACP5) and P_i_ transporters (Pht1,4) involved in remobilization and re-translocation of P_i_ from RNA and other organic P compounds in senescing organs (**Figures 1** and **2**), during programmed cell death and also in response to P_i_ limitation (Thibaud et al., 2010; Nagarajan and Smith, 2011). Accordingly, increased ethylene responsiveness has been implicated also in the formation of lysigenic aerenchyma in P_i_-deficient maize roots (He et al., 1992, 1994) as a strategy for P_i_ recycling.

## Cluster Roots

The formation of cluster roots (CR) belongs to the most specialized adaptations for mobilization of sparingly soluble P_i_ sources in soils (**Figure 2**). Although CRs have been detected in various plant families such as Proteaceae, Casuarinaceae, Mytaceae, Fabaceae, and others, white lupin so far represents the best-characterized model plant with respect to regulatory aspects of CR development and CR function. CRs are bottlebrush like structures formed by short second-order laterals with determinate growth and densely covered with root hairs (Dinkelaker et al., 1995; Neumann and Martinoia, 2002). Thereby, the largely increased surface area enables a concentrated release of organic metal chelators (citrate, malate, phenolics), ectoenzymes (acid phosphatases), protons and reductive changes in the rhizosphere, mediating the mobilization of sparingly soluble soil phosphates but also other nutrients, such as Fe, Mn, Zn, and Mo (Gardner et al., 1983; Dinkelaker et al., 1997, Neumann and Römheld, 2007). In the past, only a few studies addressed a possible involvement of ethylene in CR development, mainly with inhibitor studies and measuring ethylene evolution from the whole root system (Gilbert et al., 2000; Zaid et al., 2003). More recently, transcriptomics and gene expression studies revealed considerable variation in the expression of genes encoding ethylene bio-synthetic enzymes (ACC oxidase, ACC synthase) during CR development (O’Rourke et al., 2013; Wang et al., 2014a,b).

In the 1.2 cm subapical root zones of first-order laterals, prior to the emergence of the second-order lateral cluster rootlets, ethylene biosynthesis genes are moderately up-regulated together with genes involved in auxin biosynthesis (YUCCA) and transport (AUX1, PIN1), synthesis of brassinosteroids, and cytokinin receptors (**Figure 3**; Wang et al., 2014a,b), in accordance with the postulated role of these hormonal factors in formation of auxin gradients required for priming of pericycle cells for induction of the lateral rootlet primordia (Fukaki and Tasaka, 2009). However, in contrast to ethylene-mediated modifications of root growth under P_i_ limitation discussed so far (including growth inhibition of the primary root and lateral root proliferation), CR formation is largely induced systemically determined mainly by the P_i_-nutritional status of the shoot (Marschner et al., 1987; Shane et al., 2003). Accordingly, induction of CRs in P_i_-deficient white lupin can be suppressed almost completely by foliar P_i_ application (Marschner et al., 1987). More recently, sucrose has been identified as important shoot-borne signal, triggering the formation of CRs (Zhou et al., 2008; Wang et al., 2015) mediated by the well-documented increased shoot-to root translocation of sucrose under P_i_ limitation (Hammond and White, 2008, 2011; Wang et al., 2015). Even in P_i_-sufficient lupin plants cultivated with P_i_ concentrations suppressive for CR formation, external application of sucrose to the rooting medium induced the formation of CRs in a concentration dependent manner to a similar or even higher extent than in P-deficient plants (Wang et al., 2015). Both, P_i_ deficiency-induced CR formation and sucrose-induced formation of CRs under sufficient P_i_ supply are completely suppressed by the ethylene biosynthesis inhibitor CoCl_2_ (Wang et al., 2014b). Moreover, in many other plant species it has been shown that external sucrose supply increases ethylene production in a concentration-dependent manner with effects on various processes, such as anthocyanin production, flowering, and fruit ripening (Philosoph-Hadas et al., 1985; Kobayashi and Saka, 2000; Jeong et al., 2010) and sucrose concentrations increased in the sub-apical root zones of first-order laterals in P-deficient white lupin (Wang et al., 2015). These findings raise the question whether sucrose as a shoot-borne signal exerts its stimulatory effects on CR formation via stimulation of ethylene biosynthesis. However, during outgrowth of the CR primordia in the juvenile (JU) stage of CR development, expression of transcripts involved in ethylene biosynthesis (ACC oxidase) and auxin synthesis and transport transiently declined, followed by a massive increase of ACC oxidase gene expression during cluster-root maturation (**Figure 3**; Wang et al., 2014a,b). This is associated with a range of metabolic and developmental modifications (Wang et al., 2014a) known to be mediated by ethylene signaling also in other plant species comprising: (i) initiation of determinate growth of the lateral rootlets by inactivation of the root tip meristem including interactions with jasmonic acid (Chacon-Lopez et al., 2011; Wang et al., 2014a); (ii) formation of long, densely spaced root hairs (Cho and Cosgrove, 2002; Neumann and Martinoia, 2002; Zhang et al., 2003); (iii) increased expression of root secretory acid phosphatase (Massonneau et al., 2001; Lei et al., 2011); (iv) a massive decline (80%) of total RNA contents (Massonneau et al., 2001) associated with up-regulation of ribonuclease genes and P_i_ transporters (**Figure 2**) involved in remobilization and re-translocation of P_i_ from RNA degradation to the young, actively growing root zones (Thibaud et al., 2010; Nagarajan and Smith, 2011; Wang et al., 2014a,b); (v) the massive RNA degradation during CR maturation results in the formation of NO as a side product.(Wang et al., 2010). Together with ethylene, NO may be involved in the induction of the FIT transcription factor as a central regulator of the coordinated Fe deficiency responses in strategy I plants (Hindt and Guerinot, 2012), which surprisingly was similarly expressed in mature CRs of white lupin even under Fe-sufficient conditions (Wang et al., 2014a) including also the up-regulation of the plasma membrane ferric reductase system (FRO2) and the FeII transporter (IRT1). Interestingly many adaptations of CRs toward improved P_i_ acquisition, such as root hair proliferation, proton extrusion, exudation of phenolic compounds with metal-chelating properties and increased ferric reductase activity at the root surface are also part of the strategy I mechanism for Fe acquisition (Neumann and Römheld, 2007). Since lupins are naturally adapted to moderately acidic soils frequently characterized by P_i_ fixation on iron amd aluminum oxides/hydroxides, the expression of mechanisms for Fe acquisition may be beneficial also for mobilization of sparingly soöuble Fe-P forms even at low soil pH, where Fe availability is usually not a problem. Consequently, in white lupin responses to Fe deficiency and to P limitation may at least partially share the same ethylene-dependent signaling pathways. Also in *Arabidopsis*, an interplay of strategies for P and Fe acquisition is suggested by increased Fe accumulation in response to P limitation (Mission et al., 2005; Hirsch et al., 2006; Ward et al., 2008) However, in contrast to white lupin, this was associated with a down-regulation of the IRT1 tansporter and increased expression of FER1 encoding a Fe storage protein (Mission et al., 2005). This was interpreted as a protective mechanism to counteract Fe toxicity. In white lupin, despite up-regulation of the strategy I mechanism for Fe acquisition, no excessive Fe accumulation was observed in response to P_i_ limitation (Wang et al., 2014a) and the mechanism to counteract Fe toxicity is yet unknown.

## Concluding Remarks

The recent knowledge on the role of ethylene in P_i_ acquisition of higher plants demonstrates that ethylene is much more than just a modulator of root growth for adaptations to facilitate spatial P_i_ acquisition. Increasing evidence points to numerous additional functions also in mechanisms for chemical P_i_ solubilization in the rhizosphere and internal P_i_ recycling. For future research activities in this context it will be important to demonstrate more in detail how ethylene is integrated into the signaling network mediating the respective P_i_ starvation responses, to identify receptors and how it interacts with other hormonal and non-hormonal regulators (e.g., auxin, jasmonic acid, brassinosteroids, cytokinins, GA3, abscisic acid, polyamines, NO, miRNAs, sucrose). Particularly interesting in this context are also interactions with mechanisms for acquisition of other nutrients as indicated, e.g., for a potential link between P_i_ acquisition and Fe acquisition in *Arabidopsis* and white lupin, which at least in case of white lupin share many similarities and even similar signaling pathways with ethyleäne as a modulator of both root growth responses and physiological adapatations for mobilization of Fe and P_i_. Meanwhile it seems to be clear that ethylene-mediated P deficiency responses are not based on one general mechanism and considerable genotypic variation exists between plant species and cultivars, which needs to be characterized more in detail for potential exploitation in breeding programs.

## Conflict of Interest Statement

The author declares that the research was conducted in the absence of any commercial or financial relationships that could be construed as a potential conflict of interest.
